# *Caenorhabditis elegans* susceptibility to *Daldinia* cf. *concentrica* bioactive volatiles is coupled with expression activation of the stress-response transcription factor *daf-16*, a part of distinct nematicidal action

**DOI:** 10.1371/journal.pone.0196870

**Published:** 2018-05-03

**Authors:** Payal Sanadhya, Patricia Bucki, Orna Liarzi, David Ezra, Abraham Gamliel, Sigal Braun Miyara

**Affiliations:** 1 Department of Entomology and the Nematology and Chemistry Units, Agricultural Research Organization (ARO), the Volcani Center, Rishon Lezion, Israel; 2 Department of Plant Pathology and Weed Research, ARO, the Volcani Center, Rishon Lezion, Israel; 3 Agricultural Engineering, Growing, Production and Environmental Engineering, Laboratory for Pest Management Research, ARO, the Volcani Center, Rishon Lezion, Israel; UMASS Medical School, UNITED STATES

## Abstract

The bionematicidal effect of a synthetic volatile mixture (SVM) of four volatile organic compounds (VOCs) emitted by the endophytic fungus *Daldinia cf*. *concentrica* against the devastating plant-parasitic root-knot nematode *Meloidogyne javanica* has been recently demonstrated in both *in vitro* and greenhouse experiments. However, the mode of action governing the observed irreversible paralysis of J2 larvae upon exposure to SVM is unknown. To unravel the mechanism underlying the anthelmintic and nematicidal activities, we used the tractable model worm *Caenorhabditis elegans*. *C*. *elegans* was also susceptible to both the fungal VOCs and SVM. Among compounds comprising SVM, 3-methyl-1-butanol, (±)-2-methyl-1-butanol, and 4-heptanone showed significant nematicidal activity toward L1, L4 and young adult stages. Egg hatching was only negatively affected by 4-heptanone. To determine the mechanism underlying this activity, we examined the response of *C*. *elegans* mutants for glutamate-gated chloride channel and acetylcholine transporter, targets of the nematicidal drugs ivermectin and aldicarb, respectively, to 4-heptanone and SVM. These aldicarb- and ivermectin-resistant mutants retained susceptibility upon exposure to 4-heptanone and SVM. Next, we used *C*. *elegans* TJ356 strain *zIs356 (daf-16*::*GFP+rol-6)*, LD1 *ldIs7* [*skn-1B/C*::*GFP + pRF4*(*rol-6*(*su1006*))], LD1171 *ldIs3 [gcs-1p*::*gfp; rol-6(su1006))]*, CL2166 *dvIs19 (gst-4p*::*GFP)* and CF1553 *muIs84 (sod-3p*::*GFP+rol-6)*, which have mutations in genes regulating multiple stress responses. Following exposure of L4 larvae to 4-heptanone or SVM, there was clear nuclear translocation of DAF-16::GFP, and SKN-1::GFP indicating that their susceptibility involves DAF-16 and SKN1 regulation. Application of 4-heptanone, but not SVM, induced increased expression of, *gcs-1*::GFP and *gst-4*::GFP compared to controls. In contrast, application of 4-heptanone or SVM to the *sod-3*::GFP line elicited a significant decline in overall fluorescence intensity compared to controls, indicating SOD-3 downregulation and therefore overall reduction in cellular redox machinery. Our data indicate that the mode of action of SVM and 4-heptanone from *D*. *cf*. *concentrica* differs from that of currently available nematicides, potentially offering new solutions for nematode management.

## Introduction

Plant-parasitic nematodes are important pathogens that affect a wide variety of plants with enormous economic losses [1]. These nematodes cause an estimated annual loss of 80 billion USD [2]. Among them, root-knot nematodes *Meloidogyne* spp. are the most devastating pests due to their widespread distribution and their broad range of target plants [3].

Prevalent methods for nematode control include the application of chemical nematicides, biological agents, and biological nematicidal compounds, such as phytochemicals from plants and compounds from microorganisms [4–7]. Chemical nematicides are the most common and effective method for controlling plant parasites. However, their use is frequently discouraged due to their high toxicity to humans and animals, adverse effects on the environment and the emergence of resistant strains [8]. Thus, development of less toxic alternatives with minimal environmental side effects is required to alleviate the damage caused by phytonematodes [9].

Fungi are extensively used as effective biological control agents against plant-parasitic nematodes [10]. *Trichoderma* spp. have been shown to have a nematophagous effect on various nematodes, such as *Meloidogyne* spp. [11–14], *Bursaphelenchus xylophilus* (Steiner) [15], *Tylenchulus semipenetrans* [16], and *Globodera rostochiensis* [17]. The soil fungus *Paecilomyces lilacinus* is an effective biological control agent against *Meloidogyne incognita* and *Meloidogyne hapla* in tomato plants [6, 7]. Several studies have demonstrated that biological nematicides may serve as sustainable substitutes for nematode control due to their high effectiveness and minimum effects on the environment and human health [18]. Apart from direct parasitism, fungi also produce biologically active volatile compounds belonging to different classes, such as alcohols, esters, ketones, acids, and lipids. For example, *Muscodor albus* emits volatiles that have nematicidal activity against *M*. *incognita* second-stage juveniles (J2s) [19]. Similarly, an inhibitory effect of volatiles emitted by *Fusarium* [20] and *Trichoderma* [21] on plant-parasitic nematodes has also been reported, but their modes of action are still obscure. The volatile organic compounds (VOCs) emitted from the yeast *Saccharomyces cerevisiae* also have a nematicidal effect on *Meloidogyne javanica* J2s [22]. In addition, many bacteria, such as *Bacillus simplex*, *Bacillus subtilis*, *Bacillus weihenstephanensis*, *Microbacterium oxydans*, *Stenotrophomonas maltophilia*, *Streptomyces lateritius*, and *Serratia marcescens* produce nematicidal VOCs that are effective against *Panagrellus redivivus* and *Bursaphelenchus xylophilus* [4].

Considering the immense potential of fungi as biocontrol agents and the search for new alternatives to control plant-parasitic nematodes, we recently isolated an endophytic fungus—*Daldinia cf*. *concentrica* from an olive tree (*Olea europaea* L.). This isolate was found to produce biologically active VOCs exhibiting antimicrobial activity against various plant-pathogenic fungi, including *Aspergillus niger*, *Alternaria alternata*, *Botrytis cinerea* and *Penicillium digitatum* [23]. GC–MS analysis of the emitted VOCs allowed the preparation of synthetic mixtures of these VOCs, which also demonstrated strong activity against phytopathogenic fungi [23].

Further study was performed with *D*. *cf*. *concentrica* culture and a synthetic volatile mixture (SVM) consisting of four VOCs emitted by the fungus: 3-methyl-1-butanol, (±)-2-methyl-1-butanol, 4-heptanone, and isoamyl acetate. The SVM demonstrated strong nematicidal activity against *M*. *javanica* J2s. It also decreased egg hatching and reduced disease symptoms on tomato plants [24]. Among the constituents of the SVM, some were already known to exert antimicrobial activities. The compounds 3-methyl-1-butanol and (±)-2-methyl-1-butanol, identified as volatile compounds produced from *S*. *cerevisiae*, *M*. *albus*, and a *Xylaria* sp., exhibit antifungal activity against different crop pathogens [22,25,26]. Similarly, 4-heptanone, identified from *Collimonas* strains, has been shown to exert antifungal activity [27]. Isoamyl acetate, produced from *Candida maltosa*, exhibits antifungal and antibacterial activity [28]. However, these compounds have not been indicated for combating plant-parasitic nematodes. Taken together, accumulating results indicate that *D*. *cf*. *concentrica* and a SVM that is similar to the fungal VOCs may serve as a reliable and sustainable alternative for management of the plant parasite *M*. *javanica*. However, knowledge concerning the mode of action of these defined VOCs on *M*. *javanica* nematodes and their effects on other free-living nematodes is lacking.

In the current study, we utilized the model nematode *Caenorhabditis elegans* as an alternative system to investigate the mode of action of a SVM made up of *D*. *cf*. *concentrica* VOCs. *C*. *elegans* is widely used to determine the mechanism of action of anthelmintic drugs against various nematodes infecting humans, animals, and plants. This is due to the similarity of its physiology and pharmacology to those of plant-parasitic nematodes, along with a short life cycle and the availability of mutants [29–34]. Our findings demonstrate that larval stages L1 and L4 and young adults (YAs) are susceptible to 4-heptanone and SVM. Direct exposure of eggs, and L1 and L4 stages to 4-heptanone and SVM inhibited locomotion, feeding, and further growth and development. Experiments with a mutant that is resistant to aldicarb (anticholinesterase compounds), carrying a hypomorphic mutation in the vesicular acetylcholine transporter [35], and a mutant resistant to ivermectin (a macrocyclic lactone), carrying functional null mutations in three genes encoding glutamate-gated chloride channels (GluCls) [36], indicated that these corresponding pathways are not required for the nematicidal activity of VOCs. We also used transgenic green fluorescent protein (GFP)-tagged stress-reporter strains of *C*. *elegans* to trace the stress pathways elicited by 4-heptanone and SVM exposure. Our findings place *C*. *elegans* as a suitable system for studying the mode of action of bioactive VOCs from *D*. *cf*. *concentrica* while excluding the mechanism involved in action of anticholinesterases and macrocyclic lactone compounds.

## Materials and methods

### *Caenorhabditis elegans* mutants

*Caenorhabditis elegans* strains were acquired from the Caenorhabditis Genetics Center (CGC) (University of Minnesota, (Minneapolis, MN, USA). N2 Bristol was used as the reference strain. *C*. *elegans* mutant strains used in this study were CB113 *unc-17(e113)* that carries a hypomorphic mutation in the vesicular acetylcholine transporter [35], and DA1316 *avr-14(ad1302); avr-15(ad1051); glc-1(pk54)*, which carries functional null mutations in three genes encoding GluCls [36]. The GFP reporter lines used were: TJ356 strain *zIs356 (daf-16*::*GFP + rol-6)* carrying a GFP reporter fused to *daf-16* to visualize DAF-16 expression, LD1 *ldIs7* [*skn-1B/C*::*GFP + pRF4*(*rol-6*(*su1006*))] carrying a GFP reporter fused to SKN-1/Nrf2 transcription factor, LD1171 *ldIs3 [gcs-1p*::*gfp; rol-6(su1006))]* study the inducible γ-glutamylcysteine synthe- tase (*gcs-1*) gene expression, CL2166 *dvIs19 (gst-4p*::*GFP)* to assess inducible glutathione S-transferase (*gst-4*) gene expression, and CF1553 strain *muIs84 (sod-3p*::*GFP+rol-6)* expressing GFP as a reporter transgene for inducible superoxide dismutase (*sod-3*) gene expression.

### Culturing and maintenance of *C*. *elegans*

*Caenorhabditis elegans* wild-type (WT) and mutant strains were cultured and maintained at 22°C on nematode growth medium (NGM) agar plates containing *Escherichia coli* (OP50) acquired from the Caenorhabditis elegans Center (CGC), University of Minnesota (Minneapolis, MN, USA). Age-synchronized populations of *C*. *elegans* were obtained by modified bleaching method [37]. NGM plates containing gravid adults were washed with 0.01 M 2-(N-morpholino) ethanesulfonic acid hydrate (MES) (Sigma-Aldrich, St. Louis, MO, USA) buffer, and freshly prepared bleaching solution (5 mL of 6% NaOCl, 5 mL of 1 M NaOH and 10 mL water) was added. The worm suspension was vortexed for 2 min then washed with 0.01 M MES buffer. The worm pellet was resuspended in bleaching solution and vortexed for 1 min followed by four washings with 0.01 M MES buffer. Eggs were kept overnight for hatching in an incubator shaker at 20°C. L1 larvae were inoculated on NGM plates with OP50 and incubated at 22°C, and L4 larvae were obtained from those plates after 28 h, YAs after 38 h. Worms at the respective stages were harvested from the plates by washing with 5 mL 0.01 M MES. These worms were washed four times with 0.01 M MES to remove the adhering bacteria before conducting assays.

### Effect of *D*. *cf*. *concentrica* culture on *C*. *elegans* viability

The methodology used in this study to analyze the effects of *D*. *cf*. *concentric* on *C*. *elegans* was adopted from a previous study by Liarzi et al. [24]. To assess the bionematicidal activity of *D*. *cf*. *concentrica* culture against *C*. *elegans*, pure culture plates of the fungus were used as follows: a plug of *D*. *cf*. *concentrica* was transferred to a 50-mm Petri plate containing 5 mL potato dextrose broth (PDB; Acumedia, Lansing, MI, USA), and incubated at 25°C for 5 days. Experiments (duplicates for each treatment, three independent experiments) were carried out in 1-L plastic boxes sealed with plastic wrap with no physical contact between the larvae and the tested volatile compounds. Therefore, the effect on larval viability could only be through the emitted VOCs. Each box contained three uncovered culture plates of *D*. *cf*. *concentrica* and five small vials (12 × 35 mm, Fisher Scientific), each containing 300 *C*. *elegans* (L1–L2) larvae suspended in 0.5 mL of 0.01 M MES. To maintain humidity in the box, we placed an additional 50-mm Petri plate containing 10 mL sterile double-distilled water. Similar boxes without culture plates served as controls. All boxes were incubated at 25°C in the dark for 2 days. The nematodes were harvested from the vials and washed with 0.01 M MES on a 30-μm filter (AD Sinun Technologies, Petach Tikvah, Israel). Filters were then transferred to 15-mL Falcon tubes with 0.9 mL 0.01 M MES. The filters with larvae were incubated for 2 h in the dark at 25°C, and viable nematodes, which actively pass through the filter, were counted using nematode-counting slides (Chalex LLC, Portland, OR, USA) under an inverted microscope (Wilovert Standard, Helmut Hund GmbH, Wetzlar, Germany).

### Study of lethal effects of VOCs on viability of different larval stages of *C*. *elegans*

The methodology used in this study to analyze the effect of VOCs and SVM on *C*. *elegans* larvae was adopted from Liarzi et al. [24]. The SVM comprised the following compounds in accordance to the previously identified VOCs: 3-methyl-1-butanol (0.1 mL, 0.92 mmole), (±)-2-methyl-1-butanol (0.1 mL, 0.93 mmole), 4-heptanone (0.2 mL, 1.43 mmole), and isoamyl acetate (0.1 mL, 0.68 mmole), at a volumetric ratio of 1:1:2:1. All compounds were of the highest available purity and procured from Sigma-Aldrich (St. Louis, MO, U.S.A). The experiments (duplicates for each treatment, three independent experiments) were carried out as above except that the fungus culture plates were replaced with individual VOCs or SVM. Five small vials (12 × 35 mm), each containing 500 eggs or 300 *C*. *elegans* larvae suspended in 0.5 mL 0.01 M MES were lined up in each box. To discriminate between effects on egg hatching but not on hatched L1 larvae, eggs were exposed for 5 h to the respective VOCs. For treatments with individual VOCs, each compound was adsorbed onto 50 mg perlite particles placed on a 50-mm Petri plate loaded with 3-methyl-1-butanol, (±)-2-methyl-1-butanol or isoamyl acetate (0.1 mL each), or 4-heptanone (0.2 mL preloaded on 100 mg perlite). The SVM (0.5 mL) was loaded on 250 mg perlite particles in a 50-mm Petri plate. The control was devoid of any VOCs. The boxes with individual VOCs, SVM and without VOCs (control) were incubated as above. The nematodes were harvested from the vials and washed with 0.01 M MES on a 25-μm filter (AD Sinun Technologies, Petach Tikvah, Israel) for L1 larvae, and 60 μm for L4 larvae and YAs. Filters were then loaded into 15-mL Falcon tubes with 0.9 mL 0.01 M MES. The nematodes that actively passed through the filter were counted using nematode-counting slides (Chalex LLC, Portland, USA) under the inverted microscope. (Wilovert Standard, Helmut Hund GmbH, Wetzlar, Germany).

### Assessment of the effect of 4-heptanone and SVM on *C*. *elegans* development

To study the effect of VOCs on the progression of *C*. *elegans* developmental stages, the eggs, and L1 and L4 larvae were inoculated on NGM plates with *E*. *coli* OP50 and exposed to 4-heptanone or SVM [38]. Experiments (duplicates for each treatment, three independent experiments) were performed in 1-L plastic boxes sealed with plastic wrap. Uncovered NGM plates containing eggs, or L1 or L4 larvae were then transferred to the boxes containing 4-heptanone (0.2 mL preloaded on 100 mg perlite) or SVM (0.5 mL preloaded on 250 mg perlite) in 50-mm Petri plates. To maintain humidity in the box, a 50-mm Petri plate with 10 mL sterile double-distilled water was added. The boxes without any VOCs served as controls. All boxes were incubated at 25°C in the dark. The effect of VOCs on eggs, and L1 and L4 larvae was observed under the microscope after 48 h exposure. Bright-field images were acquired with a Nikon Eclipse 80i microscope equipped with a Nikon Y-TV55 camera. All pictures were taken at 20X magnification. Nematodes that were straight or curved and immotile were considered dead.

### Comparative effects of VOCs, aldicarb and ivermectin on *C*. *elegans* viability

Stock solutions of aldicarb (Sigma-Aldrich, St. Louis, MO, USA) and ivermectin (Sigma-Aldrich, St. Louis, MO, USA) were prepared in 70% ethanol and 100% dimethyl sulfoxide (DMSO), respectively, and stored at -20°C. Working solutions of these drugs were prepared in 0.01 M MES. To test whether the effect of the VOCs on nematode susceptibility was similar to those of aldicarb and ivermectin, assays were conducted with L4 larvae of *C*. *elegans* strains CB113 (resistant to aldicarb), DA1316 (resistant to ivermectin), and WT N2 Bristol. Experiments were carried out as described above. Selected concentrations of the drugs were added directly to the nematode-containing vials, and larvae suspended in 0.01 M MES served as controls. For the ivermectin and aldicarb experiments, nematodes in the control treatment were also supplemented with equal volumes of DMSO and 70% ethanol used for treatments. At the end of the incubation, nematode viability was assessed as described above, and bright-field images were captured with a Nikon Eclipse 80i microscope equipped with a Nikon Y-TV55 camera for all treatments.

### GFP expression and quantification in *C*. *elegans*–GFP reporter lines

The L4 larvae of transgenic strains TJ356, LD1, LD1171, CL2166, and CF1553 were treated with 4-heptanone and SVM as described above for 24 h. No VOCs were added to the controls. After incubation, live TJ356, LD1171, CL2166, and CF1553 worms were mounted on slides and images were captured with a Leica DMLB epifluorescence microscope (Leica Microsystems GmbH, Wetzlar, Germany) equipped with a Nikon DS-Fi1 camera. The fluorescence images for SKN-1::GFP in LD1 strain were acquired by an Olympus BX63 microscope coupled to *cellSens Dimension* Imaging Software. All pictures were captured using identical exposure settings at 20X magnification for each strain. The quantification of fluorescence intensity was done by ImageJ software.

### Statistical analysis

Statistical analyses were performed with the JMP 10.0.1 software package (SAS Institute, Cary, NC, USA). All experiments were repeated three times with similar results, and the results of one representative experiment are presented. Data for each independent experiment were analyzed by comparison of all means using Tukey–Kramer test with α level of 0.05. Nonparametric data were analyzed using the Mann-Whitney U test. The results of nuclear localization quantification values in *C*. *elegans* strain TJ356 were analyzed using the independent sample student t-test. Means were considered statistically significant at p ≤ 0.001.

## Results

### *Daldinia cf*. *concentrica* culture demonstrates nematicidal action against *C*. *elegans*

**Fig 1:**
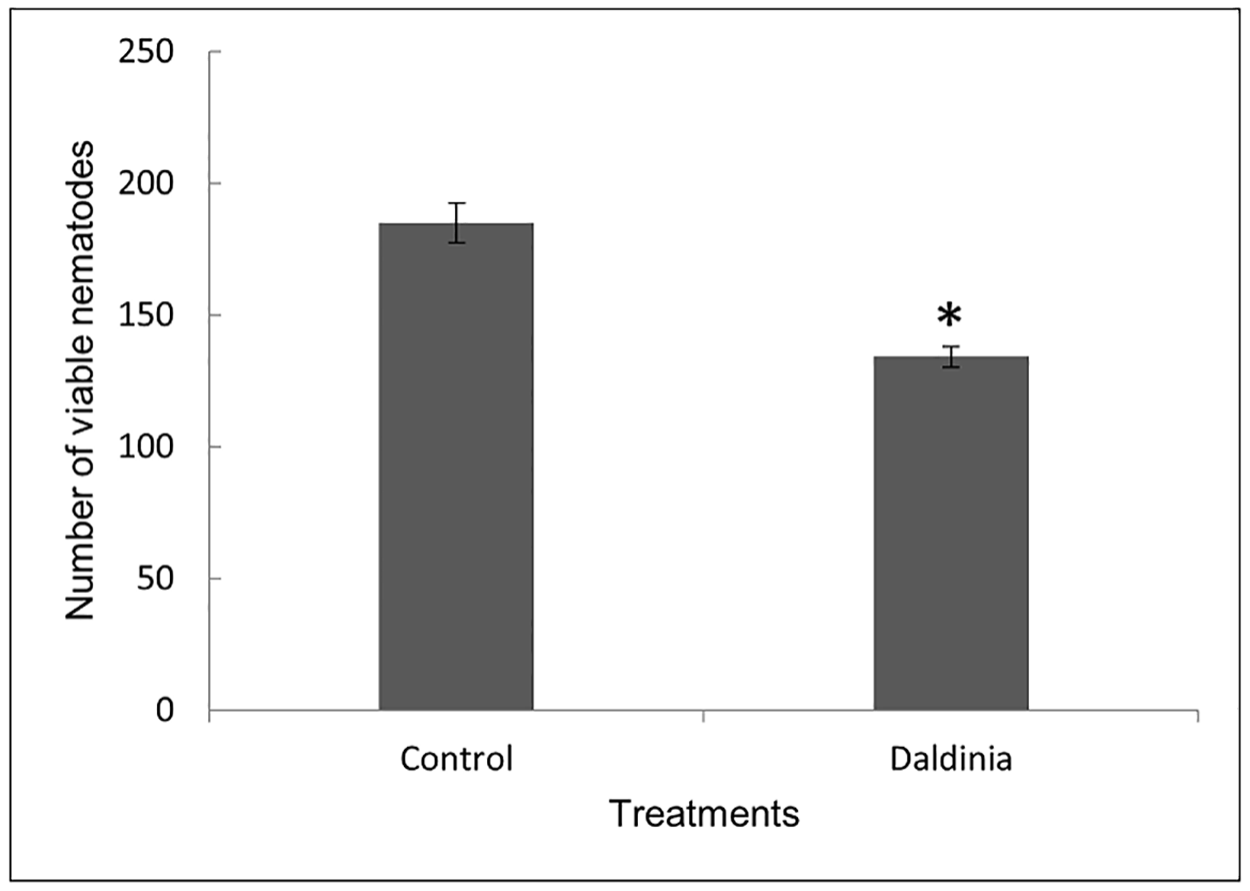
Effect of *D*. *cf*. *concentrica* culture on L1–L2 larvae of *C*. *elegans*. L1–L2 larvae of WT *C*. *elegans* were exposed for 48 h to three 50-mm diameter *D*. *cf*. *concentrica* culture plates. The viable nematodes passed through the 30-μm sieve and were counted. Ten replicates were used for each treatment with 300 nematodes per replicate. Graphs are representative of three independent experiments. Significant differences (as indicated by independent Student’s t-test) between control and *Daldinia* treatments are denoted by an asterisk (*P* ≤ 0.05).

The experiment was carried out to study the direct effect of VOC’s emitted from *Daldinia* culture plate on *C*. *elegans* L1-L2 stage larvae. Exposure of L1–L2 stages of *C*. *elegans* to the volatiles emitted by three culture plates of *D*. *cf*. *concentrica* significantly reduced *C*. *elegans* viability by 28% compared to controls (Fig 1).

### Effects of VOCs and SVM on *C*. *elegans* egg hatching

**Fig 2:**
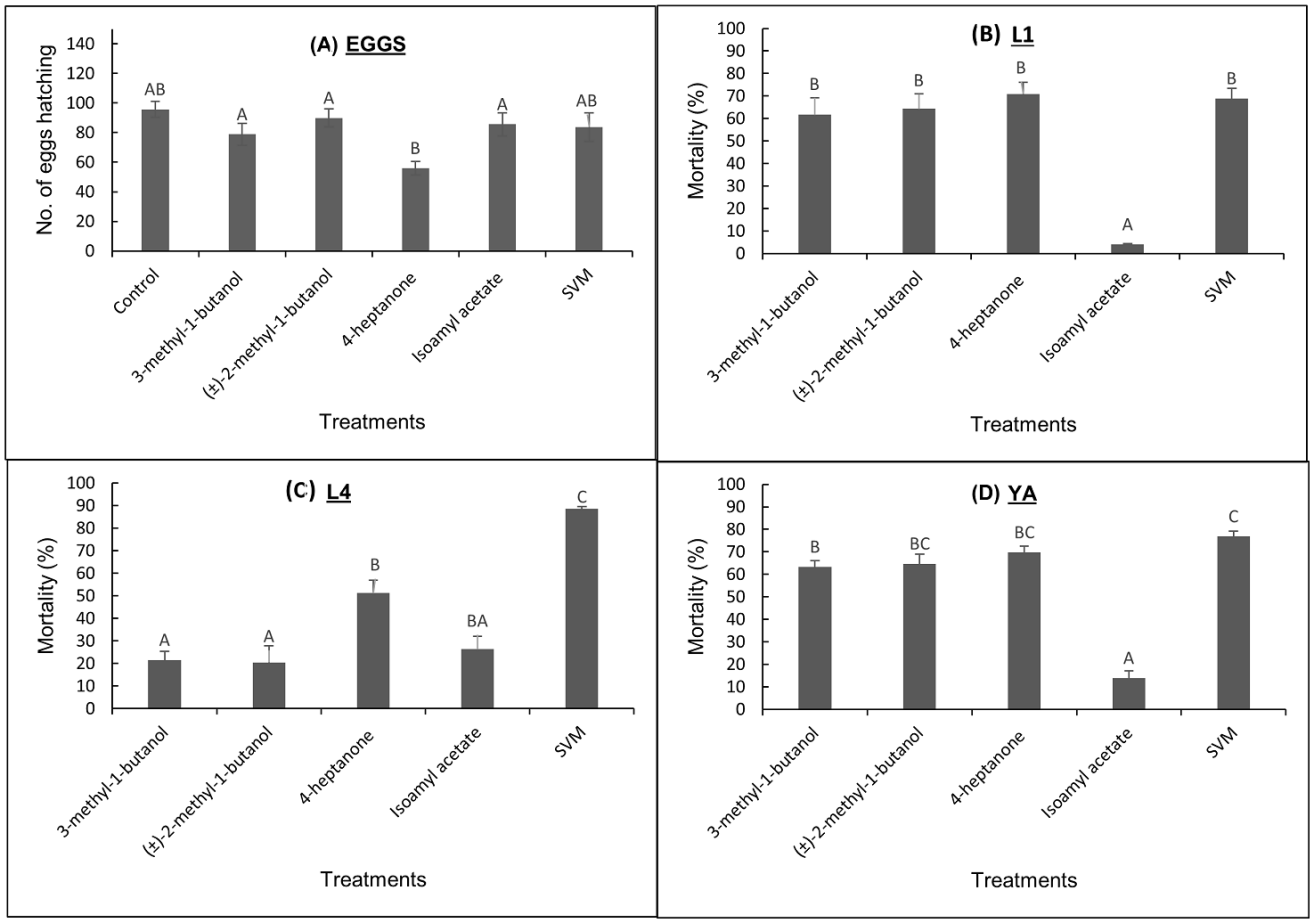
Effects of individual VOCs and SVM on WT *C*. *elegans*. (A) Eggs, (B) L1, (C) L4 and (D) YA larval stages. Eggs or nematodes were exposed to 3-methyl-1-butanol (0.1 mL, 0.92 mmole), (±)-2-methyl-1-butanol (0.1 mL, 0.93 mmole), 4-heptanone (0.2 mL, 1.43 mmole), isoamyl acetate (0.1 mL, 0.68 mmole), or up to 0.5 mL of SVM. The viable nematodes were passed through sieves and counted. Data on the y-axis are presented as no. of eggs hatching (A) and as percent mortality relative to controls (mean ± SE) (B-D). Graphs are representative of three independent experiments. Pairwise comparison of the means was performed with analysis of variance followed by post-hoc Tukey–Kramer multiple comparison test. Different letters indicate significantly different values (*P* ≤ 0.05).

The effect of VOCs and SVM on hatching of *C*. *elegans* eggs depended on the VOC examined. Hatching of *C*. *elegans* eggs after exposure to VOCs and SVM was not much affected by compounds 3-methyl-1-butanol, (±)-2-methyl-1-butanol, isoamyl acetate, or SVM (Fig 2A). However, exposure of the eggs to 1.43 mmole 4-heptanone significantly reduced their hatching percentage by 42% (Fig 2A).

### Effects of VOCs and SVM on *C*. *elegans* L1, L4 and YA viability

In a previous study, complete SVM, as well as each of its four constituents, significantly reduced the viability of *M*. *javanica* J2s. However, SVM and the constituent 4-heptanone elicited the most significant effect [24]. We therefore examined the activity of individual VOCs and SVM on different larval developmental stages of *C*. *elegans*. As shown in Fig 2B and 2D, SVM and its constituents, except isoamyl acetate, significantly reduced the viability of L1 and YA stages. The compounds 3-methyl-1-butanol, (±)-2-methyl-1-butanol and 4-heptanone reduced the viability of L1 larvae by 61%, 64%, and 70%, respectively, which was similar to the effect of the complete SVM (68%) (Fig 2B). However, 3-methyl-1-butanol (22%) and (±)-2-methyl-1-butanol had a comparatively weaker effect on the viability of L4 larvae (21%) compared to 4-heptanone (51%) and SVM (89%) (Fig 2C). Exposure of *C*. *elegans* YAs to 3-methyl-1-butanol, (±)-2-methyl-1-butanol, 4-heptanone and SVM resulted in pronounced reductions in viability, by 63%, 65%, 70% and 77%, respectively (Fig 2D). These results suggest that similar to the data obtained with *M*. *javanica* J2s [24], the complete SVM and the compound 4-heptanone are active against all developmental stages of *C*. *elegans*. We therefore continued with 4-heptanone and the complete SVM to elucidate the mechanism of nematicidal action of these anthelmintics, using mutant strains of *C*. *elegans*.

### Effects of VOCs on *C*. *elegans* development

**Fig 3:**
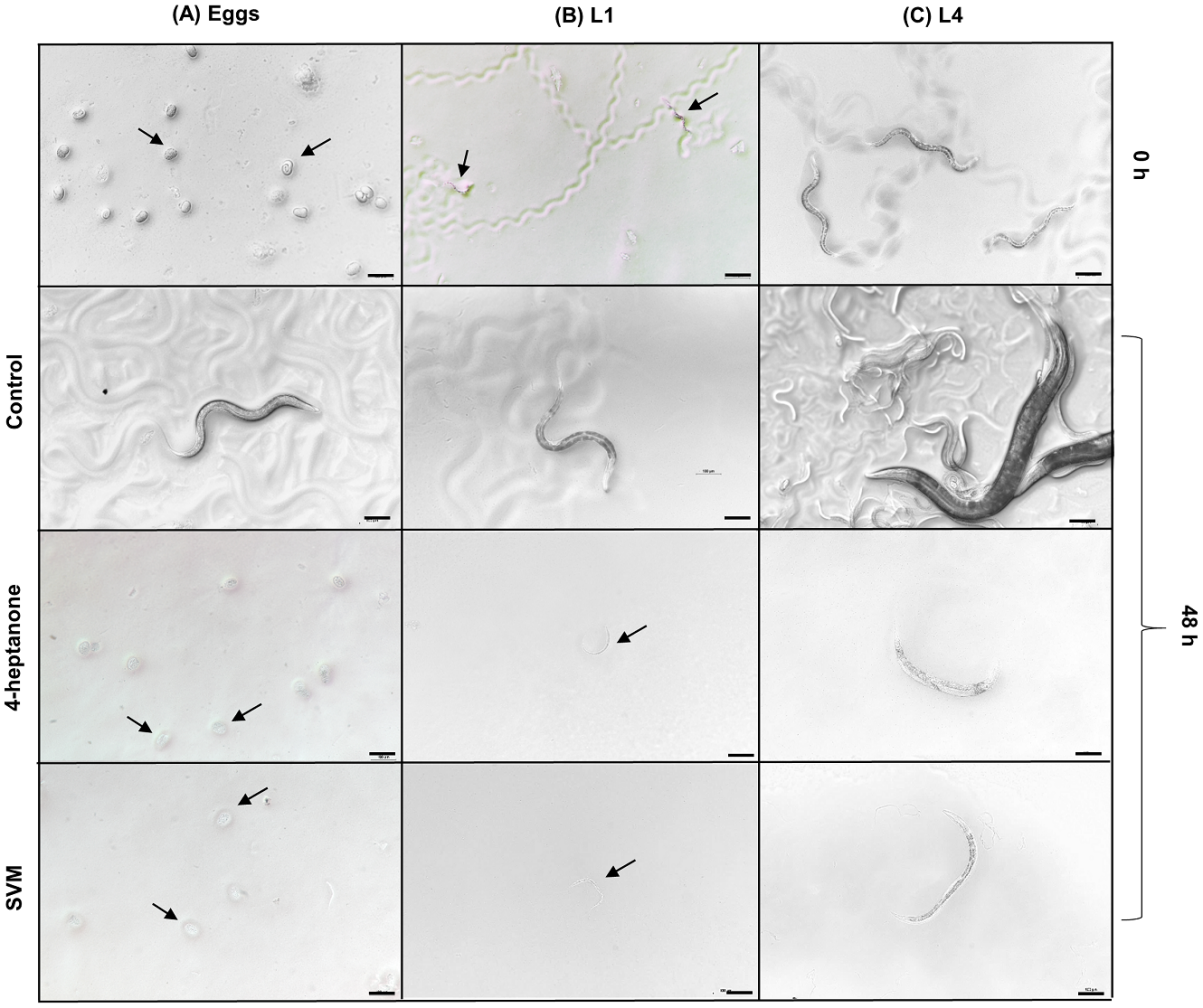
Effects of 4-heptanone and SVM on *C*. *elegans* development. Representative bright-field images of (A) eggs, (B) L1 and (C) L4 larvae on an *E*. *coli* lawn exposed to 4-heptanone (0.2 mL, 1.43 mmole) or SVM for 48 h. Scale bar = 100 μm.

To study the effects of VOCs on progression of *C*. *elegans* development, we treated different larval stages in the presence of food. Exposure of eggs to 4-heptanone and SVM resulted in inhibition of hatching after 48 h. In treatment with 4-heptanone only 8.9% hatching was observed in comparison to 82% in control. In addition, the hatched larvae did not progress beyond L1 stage after 48 h exposure to 4-heptanone. The unhatched eggs displayed refringent granular morphology instead of L1 larvae (A). Exposure of L1 larvae resulted in their growth inhibition; they were arrested at the L1 stage and were all dead within 48 h (Fig 3B). No adult worms were present among the L4 worms treated with 4-heptanone or SVM. The body of the treated nematodes was severely disorganized, making it difficult to differentiate the internal structures (Fig 3C). L1 and L4 worms were immobilized soon after transfer, as evidenced by the trail marks of the larvae on the bacterial lawn. In control plates with L4 worms, after 48 h the population consisted of different larval stages with adults and eggs. Results revealed arrested growth in L1 and L4 larvae, indicating that exposure of the nematodes to VOCs deters locomotion and feeding, and inhibits further growth and development [39].

### Comparative study of the effects of 4-heptanone and SVM with aldicarb and ivermectin nematicides on L4 viability in WT and mutant lines

**Fig 4:**
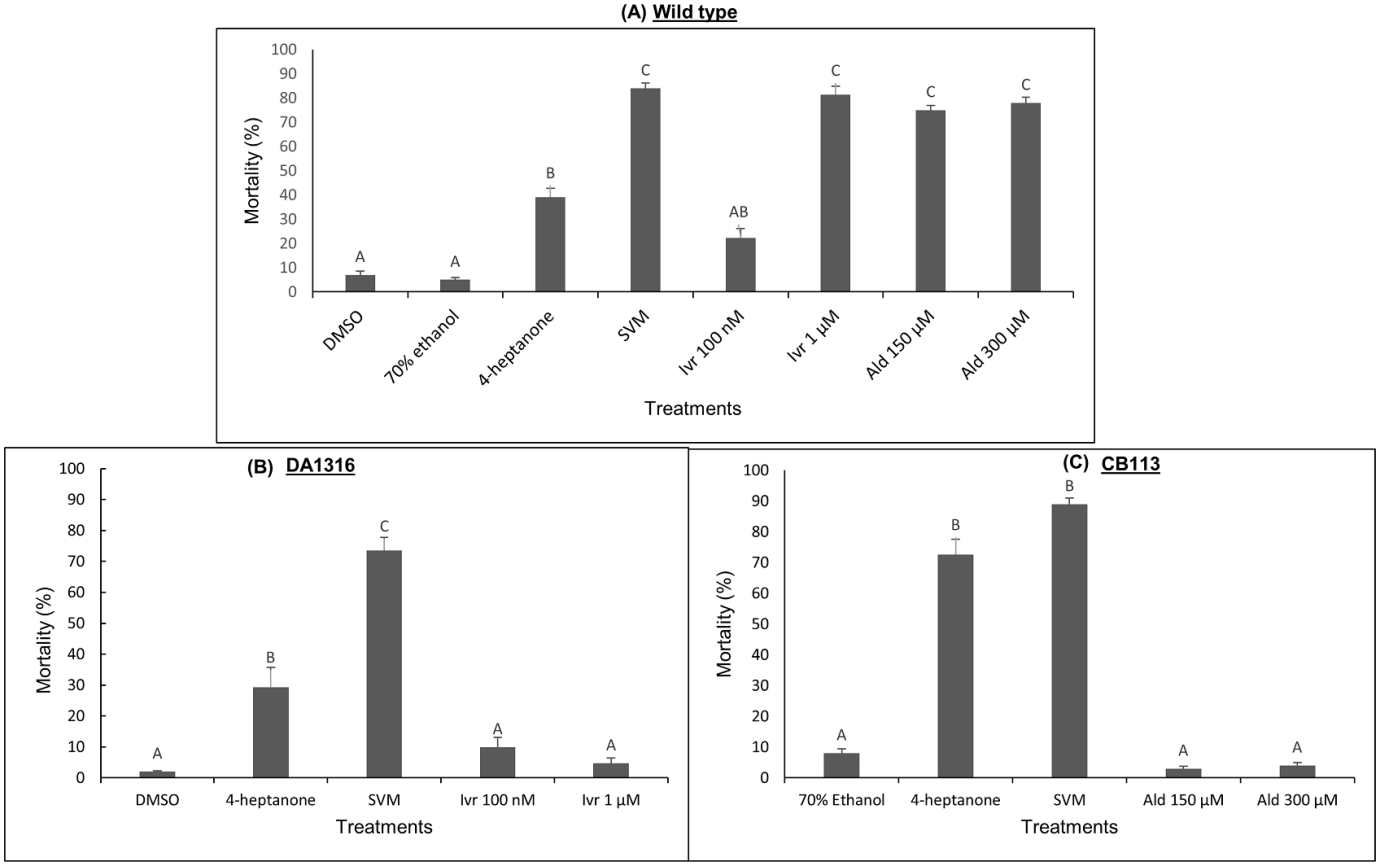
Comparison of the effects of 4-heptanone, SVM, ivermectin (Ivr) and aldicarb (Ald) on WT and mutant strains of *C*. *elegans*. (A) WT, (B) ivermectin-resistant mutant DA1316, (C) aldicarb-resistant mutant CB113. L4 nematodes were suspended in 0.5 mL 0.01 M MES in vials lined up in a sealed 1-L box. The indicated concentration of the respective drug was added to the vials containing nematodes. The nematodes were harvested after 2 days, passed through a sieve and counted. Data on the y-axis are presented as percent (mean ± SE) mortality with respect to controls. There were 10 replicates for each treatment, with 300 nematodes per replicate. Graphs are representative of three independent experiments. Pairwise comparison of the means was performed by analysis of variance followed by post-hoc Tukey–Kramer multiple comparison test. Different letters indicate significantly different values (*P* ≤ 0.05).

We investigated whether our VOCs confer their nematicidal effect by inhibiting GluCls or acetylcholine transporter, which are targets of the well-known nematicidal drugs ivermectin and aldicarb, respectively. Ivermectin is a macrocyclic lactone that is commonly used as an anthelmintic drug in both animals and humans. It binds irreversibly and activates GluCls in muscle and nerve cells, leading to paralysis of somatic and pharyngeal muscles [40,41], which leads to reduced feeding and nematode death [42]. Therefore, we examined the susceptibility of the ivermectin-resistant strain DA1316 *avr-14(ad1302); avr-15(ad1051)*; *glc-1(pk54)* to the VOCs. As shown in Fig 4, 1 μM ivermectin significantly reduced the viability of L4-stage larvae of the WT (Fig 4A) relative to those of the DA1316 nematodes (Fig 4B). In contrast, there was a significant reduction in the viability of both the WT and DA1316 mutant when treated with 4-heptanone (39% and 31%, respectively) or SVM (84% and 74%, respectively) (Fig 4A and 4B). This suggests that the mode of action of 4-heptanone and SVM differs from that of ivermectin.

Next, we investigated the effect of VOCs on the anticholinesterase activity that is required for nematode nervous system function. We used the aldicarb-resistant strain CB113 *unc-17(e113)*, which contains a hypomorphic mutation in the vesicular acetylcholine transporter, resulting in lower sensitivity to the effects of the anticholinesterase inhibitor aldicarb. *C*. *elegans* symptoms upon treatment with aldicarb include contraction of the body-wall muscles and shortening of the body, due to increased concentration of acetylcholine in the neuromuscular junctions [43] and eventually, irreversible paralysis and death of the nematode [44]. As shown in Fig 4, exposure of WT and CB113 strain L4 larvae to 150 and 300 μM aldicarb resulted in severely reduced viability of the WT worms (75–78%) at both concentrations (Fig 4A), but did not affect viability of the CB113 strain (Fig 4C). However, reduction in L4 larval viability occurred in both WT and CB113 strains upon treatment with 4-heptanone (WT, 39% and CB113, 72%) and SVM (WT, 84% and CB113, 89%) (Fig 4C). This implies that, similar to ivermectin, the mechanism by which 4-heptanone and SVM reduce *C*. *elegans* viability differs from that of aldicarb. To study phenotypic alterations upon drug application, we also examined L4 larvae of WT (S1A Fig), DA1316 (S1B Fig), and CB113 (S1C Fig) strains under the microscope following exposure to 4-heptanone, SVM or drugs (ivermectin or aldicarb). WT exposure to 4-heptanone induced constriction of the body-wall muscles at the circumference and a shrinking phenotype, leading to larval paralysis and death (S1A Fig). Whereas both 4-heptanone and aldicarb elicited larval death (S1A Fig), differences in the mode of hypercontraction and body length imply that they operate through distinct modes of action. In contrast, treatment of nematodes with SVM and ivermectin did not show any differentiable phenotypic characteristics. These results suggest that 4-heptanone and SVM may have different mechanisms of action, which do not involve activation of GluCls or anticholinesterase inhibition. These observations were in agreement with our sieve test results (Fig 4).

### Studying stress pathways induced by 4-heptanone and SVM using stress-reporter *C*. *elegans* lines

**Fig 5:**
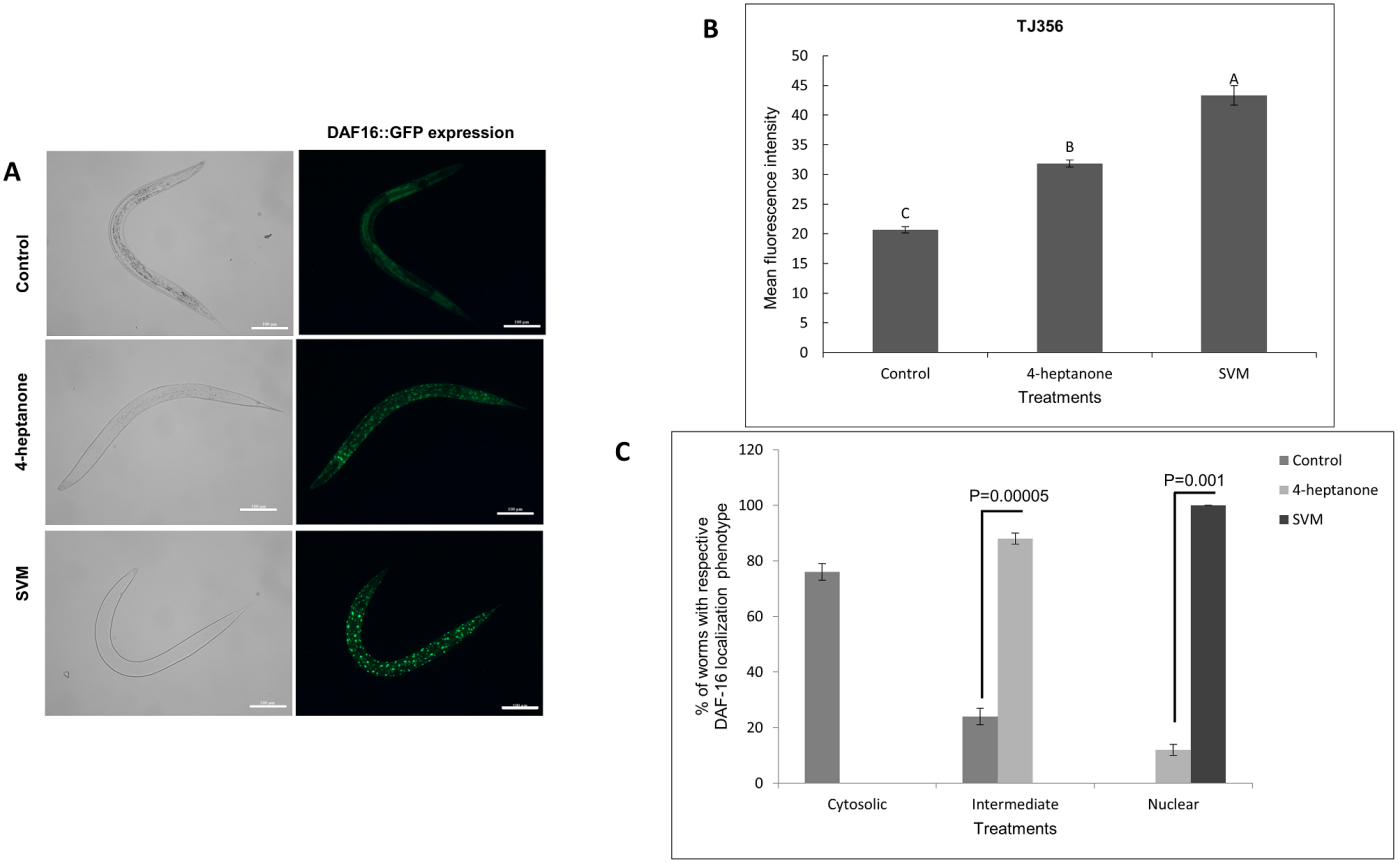
Effects of 4-heptanone and SVM on nuclear localization of DAF-16::GFP in *C*. *elegans* strain TJ356. L4 larvae from strain TJ356 were treated with 4-heptanone or SVM for 24 h. Representative images of DAF-16::GFP expression in (A) control TJ356 worms and worms exposed to 4-heptanone, and SVM. Three independent experiments with at least 10 nematodes per group were performed. Scale bar = 100 μm. (B) Quantification of DAF- 16::GFP. The y-axis denotes the average number of pixels in TJ356 nematodes measured after the treatment. Mean expression ± SE from 10 nematodes is shown. Pairwise comparison of the means was performed by analysis of variance followed by post-hoc Tukey–Kramer multiple comparison test. Different letters indicate significantly different values (*P* ≤ 0.05). (C) Effect of 4-heptanone and SVM on sub cellular distribution of DAF16::GFP. Nuclear translocation of DAF16::GFP was examined in approximately 50 worms for each condition. The worms were scored as cytosolic, intermediate, and nuclear, on the basis of localization of the DAF-16::GFP. Data are presented as mean ±SEM of the percentages of each phenotype.

In order to study whether these VOCs were inducing an oxidative stress and alter the expression of stress resistance genes, the expression of DAF-16 and SKN- 1 and their well known downstream genes *sod-3*, *gst-4* and *gcs-1* was studied. DAF-16 is an ortholog of the FOXO (forkhead box class O) family of transcription factors. This primary transcription factor is involved in the regulation of multiple stress responses, longevity, and various developmental and metabolic pathways [45–48]. Under normal conditions, DAF-16 is localized in the cytosol. However, under unfavorable conditions, such as starvation, heat, oxidative stress, hampered growth or reproduction, or when cellular repair and maintenance are required, DAF-16 moves to the nucleus. There, it elicits the transcription of a plethora of genes involved in the stress response, metabolism, and longevity [49]. Henderson and Johnson [45] showed that both mild and severe heat stress triggers nuclear translocation of DAF-16. To determine whether DAF-16 is involved in the nematode response to 4-heptanone and SVM, we examined the subcellular localization of this transcription factor using the transgenic *C*. *elegans* strain TJ356. In this strain, the reporter GFP is fused with the *daf-16* gene, thereby enabling localization of DAF-16 expression in the nematode [45]. As shown in Fig 5, control L4 larvae revealed predominantly low and diffused GFP expression (Fig 5A). However, a moderate translocation of DAF-16::GFP to the nuclei was observed in control worms as well, it might be speculated that lack of available food might induce some stress resulted in nuclear localisation. In contrast, exposure of L4 larvae to either 4-heptanone or SVM resulted in pronounced translocation of DAF-16 from the cytosol to the nucleus (Fig 5A). These treatments also induced the expression of the transcription factor, as indicated by a statistically significant increase in fluorescence intensity (Fig 5B). Interestingly, the effect achieved by SVM was more prominent than that by 4-heptanone, with significantly increased nuclear translocation (Fig 5C) and higher fluorescence intensity.

**Fig 6:**
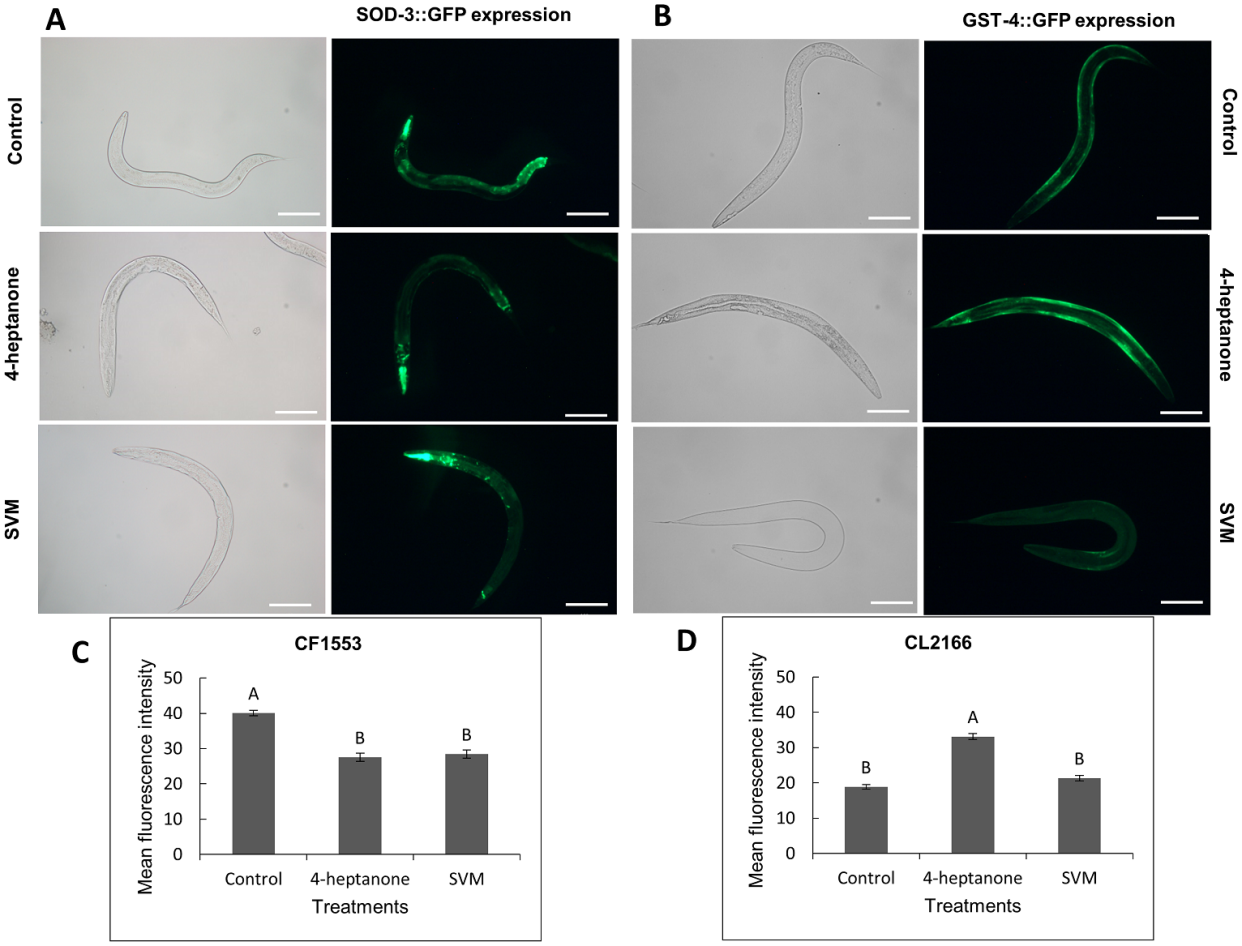
Effects of 4-heptanone and SVM on the expression of SOD-3 and GST-4 in *C*. *elegans* strain CF1553 (*sod-3*::GFP) and CL2166 (*gst-4*::GFP). L4 larvae was treated with 4-heptanone or SVM for 24 h. Representative images showing (A) *sod-3*::GFP and (B) *gst-4*::GFP expression in control or 4-heptanone and SVM-treated nematodes. Three independent experiments with at least 10 nematodes per group were performed. Scale bar = 100 μm. Quantification of (C) *sod-3*::GFP and (D) *gst-4*::GFP expression. The y-axis denotes the average number of pixels in L4 nematodes measured after the treatment. Mean expression ± SE from 10 nematodes is shown. Pairwise comparison of the means was performed by analysis of variance followed by the post-hoc Tukey–Kramer multiple comparison test. Different letters indicate significantly different values (*P* ≤ 0.05).

The excessive generation of intracellular reactive oxygen species (ROS) by external stimuli affects the redox defense mechanism, which causes oxidative stress in organisms. Antioxidant balance inside the cells is mediated through the redox defense mechanism by regulating the expression of different antioxidant enzymes [50]. SOD-3 and GST-4 are major antioxidant enzymes implicated in the oxidative stress response and regulating ROS levels [51]. SOD-3 catalyzes the conversion of superoxide radicals to hydrogen peroxide and oxygen [52]. GST-4 is a key detoxification enzyme involved in the phase II metabolic response [53–55]. Therefore, to determine whether exposure to 4-heptanone or SVM induces the oxidative stress response in *C*. *elegans* L4 larvae, we examined the expression levels of SOD-3 and GST-4 in transgenic strains CF1553 and CL2166, respectively. As shown in Fig 6, treatments with either 4-heptanone or SVM induced a significant decline in overall fluorescence intensity (Fig 6A), indicating reduction in SOD-3 expression in CF1553 nematodes relative to untreated controls (Fig 6A). In contrast, exposure of CL2166 to 4-heptanone resulted in significantly increased GFP expression associated mainly with the body-wall muscle, 75% higher than that in the control nematodes (Fig 6B and 6D). Interestingly, treatment with SVM had no significant effect on the fluorescence intensity of CL2166 nematodes (Fig 6B and 6D). This implies that only 4-heptanone elicits oxidative stress in *C*. *elegans* L4 larvae via the phase II detoxification pathway.

**Fig 7:**
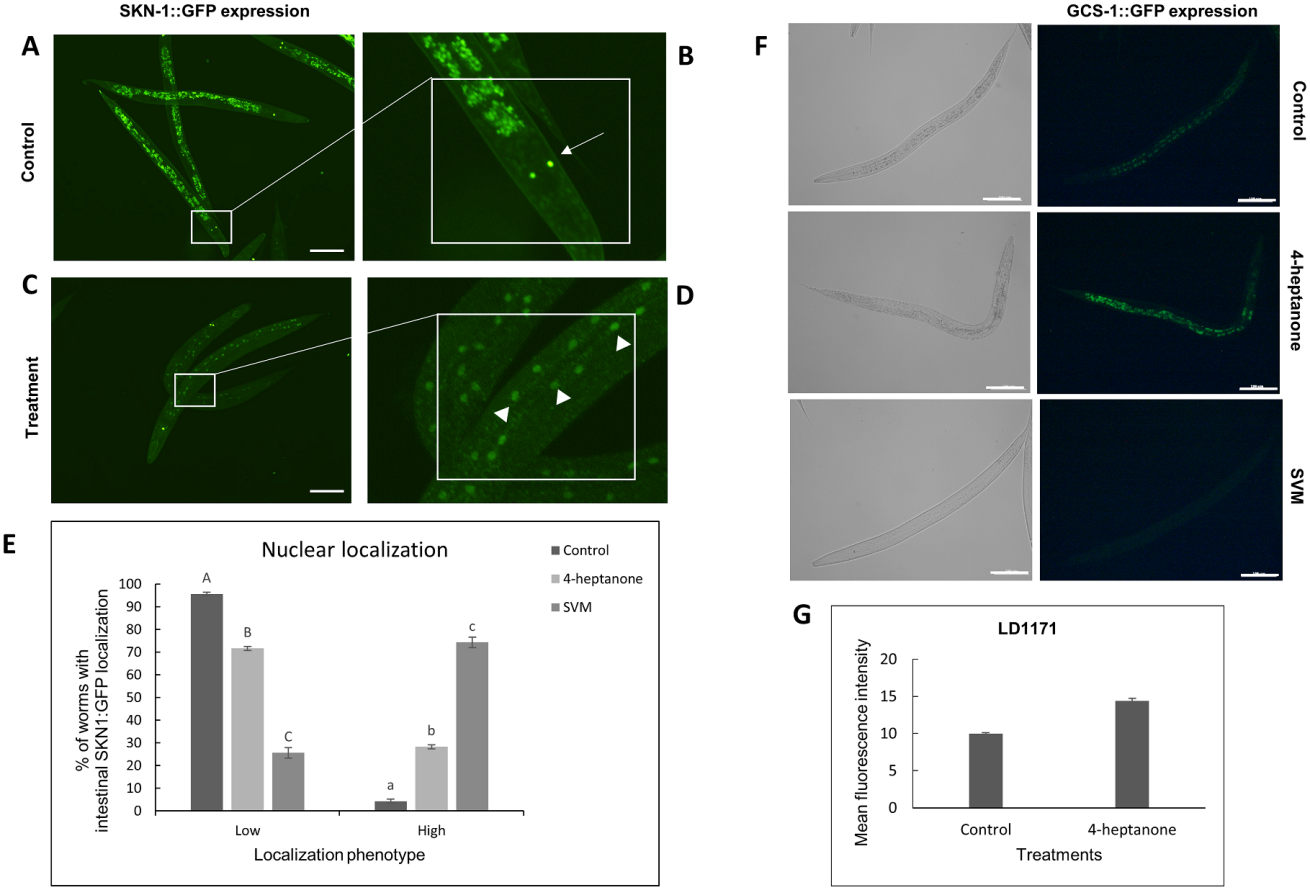
Effects of 4-heptanone and SVM on the expression of SKN-1::GFP and *gcs-1*::GFP in *C*. *elegans* strain LD1 and LD1171. L4 larvae was treated with 4-heptanone or SVM for 24 h. Representative images showing (A) SKN-1::GFP expression in control nematodes (B) Magnified view of the boxed region showing the ASI neurons (line arrow) in head region of control nematode (C) SKN-1::GFP localization in VOC treated nematodes (D) Magnified view of the boxed region in treated nematodes showing expression of SKN-1::GFP in intestinal nuclei (indicated by solid white triangles) (E) Effect of 4-heptanone and SVM on nuclear localization of SKN-1::GFP. Nuclear localization of SKN-1::GFP was scored as “Low", "Medium" or "High”. “High” refers to a strong GFP signal observed in more than 15 intestinal nuclei, "Medium" refers to nuclear GFP signals detected in 5–15 intestinal nuclei and "Low" indicates that SKN-1::GFP is hardly detectable in the intestinal nuclei. Data are presented as mean ±SEM (n = 50 worms per condition) of the percentages of each phenotype One way ANOVA and Tukey–Kramer multiple comparison test were performed independently for each localization phenotype. Different letters indicate significantly different values (*P* ≤ 0.05). (F) *gcs-1*::GFP expression in control or 4-heptanone and SVM-treated nematodes. Three independent experiments with at least 10 nematodes per group were performed. Scale bar = 100 μm. (G) Quantification of *gcs*-1::GFP expression. The y-axis denotes the average number of pixels in L4 nematodes measured after the treatment. Mean expression ± SE from 10 nematodes is shown. Mann-Whitney test indicated that fluorescence intensity in LD1171was significantly higher for 4-heptanone treatment when compared to control (p<0.001).

The SKN-1/Nrf2 transcription factor regulate the transcription of multiple genes involved in xenobiotics detoxification and antioxidant response in nematodes and mammals [56]. GCS-1 is a key enzyme involved in glutathione (GSH) synthesis and expressed at low levels in normal conditions. During oxidative stress its expression is elevated in the intestine in an SKN-1-dependent manner [57]. The regulation of GCS-1 is governed exclusively by SKN-1, while GST-4 is modulated by both DAF-16 and SKN-1 [58]. Therefore to ascertain the role of SKN-1 in VOCs mediated anthelmintic activity we assessed the expression of SKN-1::GFP and *gcs-1*::GFP reporter strains. While in control worms a low number of worms have shown high nuclear SKN-1:GFP accumulation in intestine; following 4-heptanone and SVM treatment 25% and 72% of treated worms respectively, have shown high nuclear localization phenotype as shown in Fig 7A–7E. A significant increase in induction of fluorescence was observed in *gcs-1*::GFP reporter strains upon exposure to 4-heptanone (Fig 7F and 7G). The *gcs-1*::GFP (Fig 7F and 7G) expression was suppressed by SVM treatment, even though treatment strongly activate SKN-1:GFP expression.

## Discussion

We investigated the mechanism of action of VOCs identified from *D*. *cf*. *concentrica* involved in anthelmintic activity, and compared it to that of known nematicides—the macrocyclic lactone ivermectin and the carbamate aldicarb. We also studied the stress-signaling pathways in *C*. *elegans* elicited by the most effective VOCs: 4-heptanone and SVM. To the best of our knowledge, this is the first demonstration of the mechanism of action governing the anthelmintic activity of VOCs found in *D*. *cf*. *concentrica* toward *C*. *elegans*.

As a first step to elucidating the mechanism underlying the anthelmintic activity of *D*. *cf*. *concentrica* through 4-heptanone and SVM [24], we verified that the fungus and VOCs affect *C*. *elegans* viability. We selected *C*. *elegans* because it is a popular model nematode that has been used to enhance our understanding of the modes of action of many anthelmintic compounds, such as levamisole, benzimidazole, emodepside, avermectin and fluensulfone [29,31–34,59]. Our results revealed a significant reduction of 28% in *C*. *elegans* viability upon exposure to the fungal culture (Fig 1). However, J2s of the root-knot nematode *M*. *javanica* seem to be much more susceptible (66.3 ± 14%) [24]. This variation may be due to differences in the two nematodes lifestyles (free-living versus root-knot nematode), which could affect their metabolism and sensitivity to external compounds.

Consistent with our previously published study [24], VOCs also exhibited an inhibitory effect on different developmental stages of *C*. *elegans* in MES buffer, except for egg hatching, where the only inhibition was obtained with 4-heptanone (Fig 2). Similarly, Bull et al. [38] observed variations in susceptibility of different *C*. *elegans* developmental stages in response to the anthelmintic drug emodepside. A possible explanation for the observed variations is that as it progresses through the developmental stages, the nematode undergoes significant morphological and anatomical changes, which may involve different pathways, thereby altering its response to signals. The compounds 3-methyl-1-butanol, (±)-2-methyl-1-butanol, 4-heptanone, and the complete SVM exhibited significant nematicidal activity against various developmental stages of *C*. *elegans*, relative to the effect of isoamyl acetate (Fig 2). Interestingly, while both 4-heptanone and SVM showed high nematicidal effects on both *C*. *elegans* (Fig 2) and *M*. *javanica* [24], the compound isoamyl acetate only significantly reduced viability of the latter. This suggests that the nematicidal effect of isoamyl acetate is nematode-dependent.

We further tested the ability of the most effective nematicidal VOCs, 4-heptanone and SVM, to affect the developmental rate of the WT *C*. *elegans*. The VOCs had toxic effects on the development of *C*. *elegans*, hindering nematode locomotion, feeding and further growth. We then provided further evidence of a nematicidal effect of the VOCs on *C*. *elegans* development, by showing that the latter is more sensitive to direct exposure to VOCs than to those present in MES buffer. This might be explained by differences in VOC accessibility and availability to nematodes.

To elucidate the mechanism underlying the nematicidal activities of 4-heptanone and SVM, we used *C*. *elegans* mutant strains. The mutant strain DA1316 carries simultaneous mutations of three genes—*avr-14*, *avr-15*, and *glc-1*—encoding GluCls, and is therefore resistant to ivermectin. This macrocyclic lactone, an anthelmintic compound, acts by activating or potentiating GluCl receptors, which leads to hyperpolarization of the neuronal membrane resulting in feeding inhibition and flaccid paralysis [60]. Another mutant strain used in this study was CB113, which is defective in synaptic-vesicle trafficking and therefore resistant to the effects of the carbamate drug aldicarb [35]. This drug blocks the enzyme acetylcholinesterase, and leads to accumulation of extracellular acetylcholine in the synaptic cleft, resulting in hypercontraction of the body, inhibition of feeding, growth and development, and eventually paralysis and death of WT nematodes. As expected, application of ivermectin or aldicarb reduced the viability of WT nematodes, while the corresponding mutant strains (DA1316 and CB113, respectively) remained highly resistant. However, both WT and mutant nematodes were susceptible to the application of VOCs (Fig 4). The observed behavior of the mutants toward the VOCs suggests that the molecular target for the VOCs’ effect is neither through the GluCls nor through acetylcholinesterase. Moreover, our finding that aldicarb and VOCs elicit different phenotypical effects on worm body length (Fig 4) indicates that their modes of action are different.

For further understanding the mechanism of action of 4-heptanone and SVM we evaluated the stress responses induced following the exposure by using GFP-tagged reporter strains for specific-stress pathway.

To further understand the mechanisms of action of 4-heptanone and SVM, we evaluated the stress responses induced following exposure to them using GFP-tagged reporter strains for specific stress pathways. We found that while 4-heptanone and SVM independently downregulated SOD-3 expression (Fig 6), their effects on DAF-16, SKN-1 GST-4 and GCS-1 differed: SVM had a stronger effect on the expression and nuclear translocation of DAF-16 (Fig 5) and SKN-1 (Fig 7) relative to 4-heptanone, whereas only the latter increased the expression of GST-4 (Fig 6) and GCS-1(Fig 7). Taken together, these results suggest that both 4-heptanone and SVM elicit a stress response, via a mechanism that involves DAF-16 and SKN-1 without implication of SOD-3, and that 4-heptanone confers oxidative stress via the phase II detoxification and SKN-1 pathway. A possible explanation for the observed higher nuclear translocation of DAF-16 and SKN-1 in SVM-exposed nematodes relative to 4-heptanone-exposed nematodes is the additive effect of other VOCs present in the mixture. We propose that the severity of the stress renders the cellular redox machinery vulnerable, thereby altering the expression of antioxidant enzymes SOD-3, GST-4 and GCS-1. These alterations in the redox system may contribute to SVM toxicity. Eventually, the worms were unable to cope with the stress and succumbed to paralysis and death. Similar to our results, there is evidence of downregulation of SOD-3 expression after treating *C*. *elegans* worms with high doses of tributyltin [61] or with CeO_2_ particle aggregates [62].

In summary, we showed that VOCs and SVM from *D*. *cf*. *concentrica* exhibit toxic effects on L1, L4, and YA stages of *C*. *elegans*. Viability studies involving ivermectin- and aldicarb-resistant mutants revealed that the mechanism of action of VOCs and SVM is distinct from that of the known anthelmintic compounds aldicarb and ivermectin. In further studies with GFP-tagged stress-reporter strains of *C*. *elegans*, we showed that 4-heptanone triggers the nuclear translocation of DAF-16 and SKN-1 as well as the expression of GST-4, and GCS-1 downstream effectors of DAF-16 and SKN-1 indicating oxidative stress imposed by 4-heptanone. High induction of DAF-16 and SKN-1 was also evidenced in worms treated with SVM, which might lead to the activation of the FOXO/DAF-16-dependent insulin-signaling and SKN-1 pathway. However, downregulation of *sod-3*, *gst-4* and *gcs-1* indicates a compromised redox system owing to the toxicity of SVM. In conclusion, our study is the first to reveal the mechanism of action underlying the anthelmintic activity of VOCs produced by the endophytic fungus *Daldinia cf*. *concentrica* in *C*. *elegans*.

## Supporting information

S1 Fig Comparative phenotype study of L4 larvae of *C*. *elegans* exposed to 4-heptanone (0.2 mL, 1.43 mmole), SVM, ivermectin (1 μM) or aldicarb (300 μM). Representative bright-field images of (A) WT C. elegans, (B) the ivermectin-resistant strain DA1316 and (C) the aldicarb-resistant strain CB113. Individual L4 larvae were microscopically observed and imaged after 48 h of treatment. Scale bar = 100 μm.
(TIF)
