# Supplementary figures and images for: Caenorhabditis elegans susceptibility to Daldinia cf. concentrica bioactive volatiles is coupled with expression activation of the stress-response transcription factor daf-16, a part of distinct nematicidal action
Source: PLoS One. 2018 May 3;13(5):e0196870. doi: 10.1371/journal.pone.0196870 (PMC5933902; doi:10.1371/journal.pone.0196870)

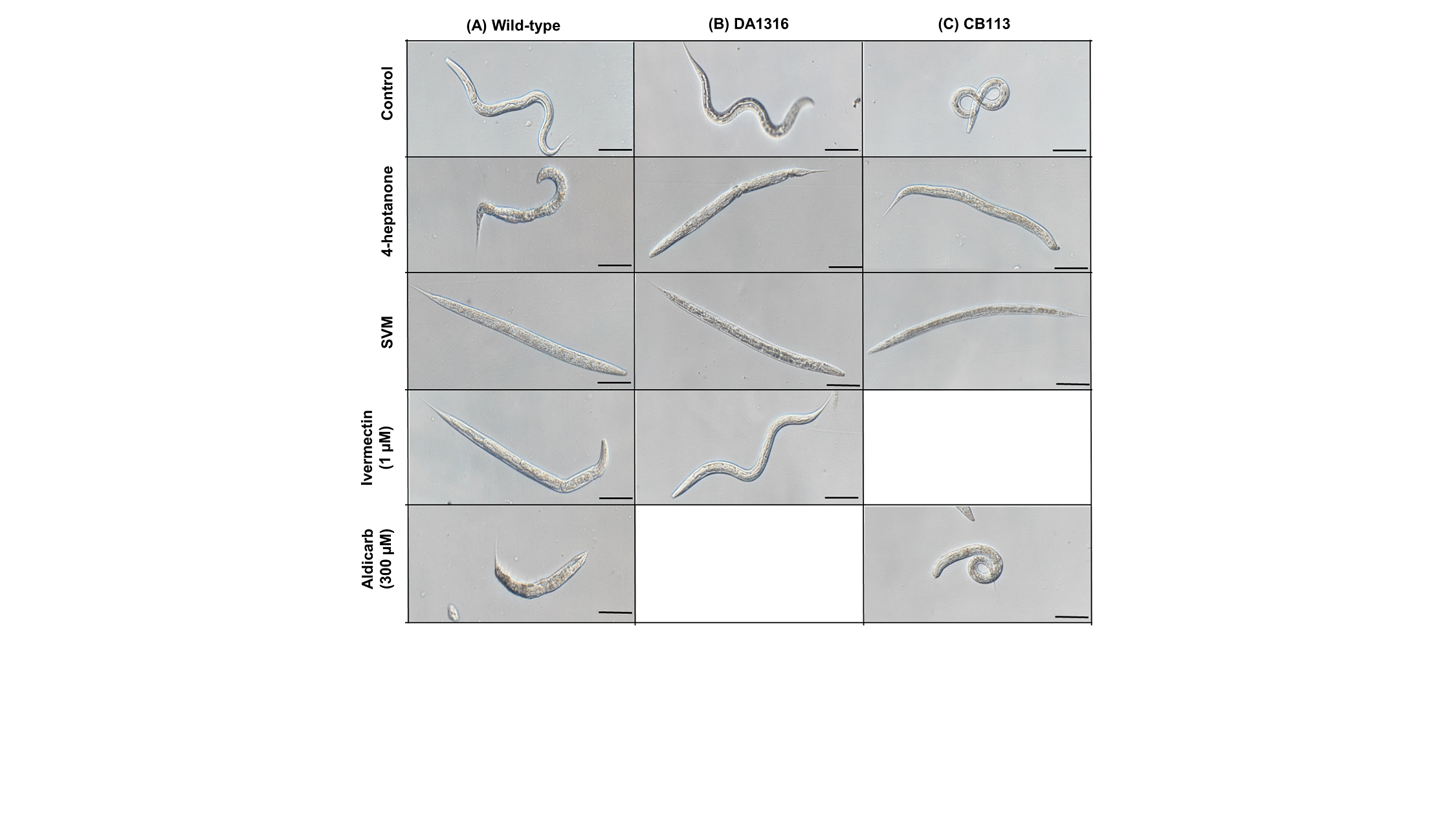

Supplement: S1 Fig — Representative bright-field images of (A) WT C. elegans, (B) the ivermectin-resistant strain DA1316 and (C) the aldicarb-resistant strain CB113. Individual L4 larvae were microscopically observed and imaged after 48 h of treatment. Scale bar = 100 μm. (TIF) [file pone.0196870.s001.tif]
